# A case of aberrant right adrenal vein anatomy

**DOI:** 10.1093/jscr/rjaf401

**Published:** 2025-06-18

**Authors:** Danielle Humphries, Bianca Marquez, Sundarachalam Pindicura, Maher Ghanem

**Affiliations:** Department of Surgery, Central Michigan University College of Medicine, 912 S. Washington Avenue, Saginaw, MI 48601, United States; Department of Surgery, Central Michigan University College of Medicine, 912 S. Washington Avenue, Saginaw, MI 48601, United States; Department of Surgery, Central Michigan University College of Medicine, 912 S. Washington Avenue, Saginaw, MI 48601, United States; Department of Surgery, Central Michigan University College of Medicine, 912 S. Washington Avenue, Saginaw, MI 48601, United States

**Keywords:** right hepatic vein, right adrenal vein, inferior vena cava, robot, adrenal cortical adenoma, adrenalectomy

## Abstract

We report a case of a robotic-assisted excision of a right adrenal gland with an aberrant right adrenal vein. A 62-year-old female presented with uncontrolled hypertension that led to the diagnosis of a right adrenal mass. Based on the clinical, imaging, and laboratory findings, the patient underwent an elective robotic-assisted right adrenalectomy. We provide an overview of the intraoperative findings and highlight the significance of utilizing a minimally invasive approach for the excision of adrenals with atypical vascular anatomy.

## Introduction

Adrenalectomies are performed for numerous pathologies, including adrenal adenomas, pheochromocytomas, and adenocarcinomas, among others [1]. Although an open approach to adrenal resection has traditionally been utilized, as technologies have advanced there has been a transition to minimally invasive techniques [2, 3]. Not only does minimally invasive surgery decrease postoperative pain and length of stay, but from a technical standpoint, it allows precise dissection, which has shown to decrease intraoperative blood loss [2, 4]. This provides a particularly important advantage when resecting adrenals with variations of typical vascular anatomy.

The anatomy of the adrenal vein is known to be quite variable [5, 6]. There may be variations in the number of adrenal veins and where it terminates. Most commonly on the left, the main adrenal vein has a longer coarse and receives the left inferior phrenic vein prior to terminating in the left renal vein [5, 7]. On the right, the adrenal vein is shorter and drains directly into the inferior vena cava, although, again, variations are common [6, 7]. Identifying and isolating the adrenal vein is an important aspect of adrenalectomy and therefore surgeons must have a comprehensive understanding of the typical anatomy of the adrenal vein and its variations to avoid intraoperative complications. This case report depicts a patient with unusual anatomy of the right adrenal vein and how adrenal resection was performed with respect to the patient’s anatomy and its associated challenges.

## Case report

**Figure 1:**
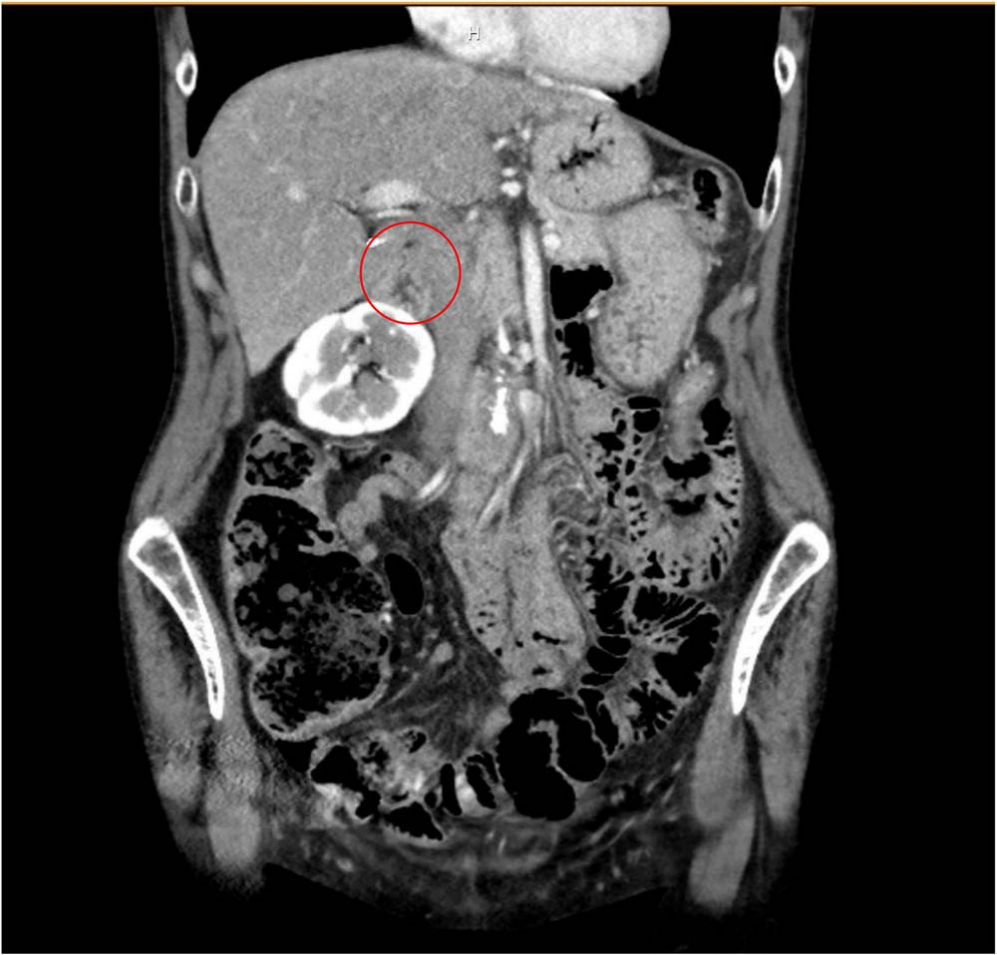
Coronal CT of abdomen and pelvis noting a 1.3 cm right adrenal mass (circle).

A 62-year-old female was found to have an incidental 1.3 cm right adrenal mass on computed tomography (CT) imaging, which was completed for suspected colitis (Fig. 1). The patient’s medical history was significant for hypertension. Following resolution of acute pathology, the patient was evaluated by endocrine surgery. During evaluation the patient denied episodic headaches and diaphoresis, weight loss, weight gain, and abdominal pain. Next, the functional status of the mass was evaluated given a history of poorly controlled hypertension despite three anti-hypertensives. The workup revealed elevated norepinephrine (1092 pg/ml), dopamine (37 pg/ml), and plasma catecholamine (1156 pg/ml). Low dose overnight dexamethasone suppression test showed slightly elevated cortisol level of 2 μg/dl. The serum renin/aldosterone, and urine metanephrine were normal. Given the concern for a functional adrenal adenoma, the patient was counseled on the risks and benefits of surgery, and it was recommended that she undergo a right adrenalectomy. Prior to surgery, the patient was evaluated by endocrinology and was pre-medicated with prazosin for 10 days followed by metoprolol.

**Figure 2:**
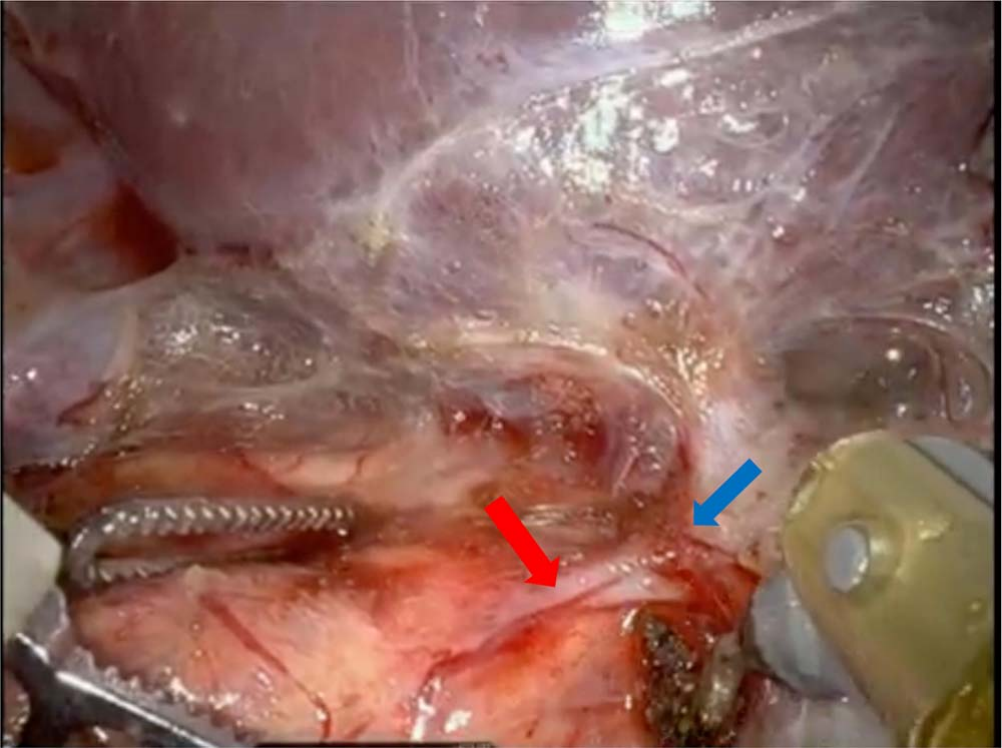
Intraoperative images show the adrenal vein (red arrow) originating from the right hepatic vein (blue arrow).

The patient was taken to the operating room and positioned right lateral decubitus after the induction of anesthesia. The peritoneum was entered supraumbilical and insufflated utilizing a Veress needle and an 8 mm robotic trocar was placed. Additional 8 mm trocars were placed along the anterior axillary line, right upper quadrant, and in the subxiphoid region. Lastly, a 12 mm trocar was placed in the left lower quadrant. The daVinci XI platform was utilized. Once the liver was retracted superiorly, the right adrenal and its associated mass were easily visible. Due to the proximity of the vena cava, careful dissection was carried along the medial side of the adrenal gland. The adrenal vein was not readily visible; thus, the lateral attachments were taken down to prevent unwanted tension on the tissue. The adrenal vein was then visualized draining into the right hepatic vein (Fig. 2). This was noted to be an unusual anatomic variation of the right adrenal vein. Meticulous dissection to allow circumferential access to the right adrenal vein and provide adequate length for clipping far from the right hepatic vein was accomplished. The adrenal vein was then clipped and divided. The adrenal gland was carefully dissected from the surrounding structures. The specimen was retrieved using an Endocatch bag.

There were no postoperative complications, and the patient was discharged on postoperative day 1 with resolution of her hypertension. The pathology revealed a 5.4 × 4 × 1.3 cm adrenal cortical adenoma.

## Discussion

In this case, the importance of identifying variant anatomy was crucial to a successful and safe adrenalectomy. The use of robotic technology allowed for improved dexterity and precision during the dissection. Early identification of the proximity of the right adrenal vein to the right hepatic vein prevented significant intraoperative hemorrhage as well. This variant of the right adrenal vein draining into the right hepatic vein has not been reported. Prior described variations of the right adrenal anatomy include posterior hepatic veins draining into adrenal veins, two right adrenal veins draining into the inferior vena cava, drainage into the lumbar vein, three adrenal veins, and two adrenal veins with one draining into the IVC and the other into the renal [6–9]. Of note, it is important to recognize that adrenal vein variations are more common on the right compared to the left [9].

A study that evaluated a robotic approach to adrenalectomy comparing patients with large tumors (5–8 cm) and left and right adrenal tumors found no difference in operative time, length of stay, conversation to open and blood loss [10]. This finding is not isolated as other studies have shown no difference in outcomes in robotic adrenalectomy when comparing right and left adrenalectomies based on size [11, 12]. Several studies have analyzed the complications between laparoscopic and robotic approaches to adrenalectomy. According to Brandao *et al.*, a meta-analysis analyzing nine studies through the years 2004–13 revealed that higher estimated blood loss and length of hospital stay were significantly less in the laparoscopic group compared to the robotic group [13]. Subsequently, in 2017, another meta-analysis again conveys a shorter stay associated with the robotic approach. However, this paper reinforces the feasibility of robotics in terms of challenging anatomy in the retroperitoneal approach [14]. For this case, the use of robotic technology was imperative to successfully recognizing and managing the aberrant adrenal vein anatomy and given studies have shown a robotic approach is safe, feasible, and not inferior to laparoscopy or open techniques, we advocate for robotic adrenalectomies.

A comprehensive understanding of adrenal vasculature and its variations is a key component of adrenalectomies for surgeons performing the operation to ensure the safety of patients.
